# Oxygen Partial Pressure Impact on Characteristics of Indium Titanium Zinc Oxide Thin Film Transistor Fabricated via RF Sputtering

**DOI:** 10.3390/nano7070156

**Published:** 2017-06-26

**Authors:** Ming-Hung Hsu, Sheng-Po Chang, Shoou-Jinn Chang, Wei-Ting Wu, Jyun-Yi Li

**Affiliations:** Institute of Microelectronics & Department of Electrical Engineering Center for Micro/Nano Science and Technology Advanced Optoelectronic Technology Center, National Cheng Kung University, Tainan 701, Taiwan; hsuminghung0121@gmail.com (M.-H.H.); changsj@mail.ncku.edu.tw (S.-J.C.); waiting31317@gmail.com (W.-T.W.); z823040@gmail.com (J.-Y.L.)

**Keywords:** indium titanium zinc oxide, thin film transistor, oxygen partial pressure

## Abstract

Indium titanium zinc oxide (InTiZnO) as the channel layer in thin film transistor (TFT) grown by RF sputtering system is proposed in this work. Optical and electrical properties were investigated. By changing the oxygen flow ratio, we can suppress excess and undesirable oxygen-related defects to some extent, making it possible to fabricate the optimized device. XPS patterns for O 1s of InTiZnO thin films indicated that the amount of oxygen vacancy was apparently declined with the increasing oxygen flow ratio. The fabricated TFTs showed a threshold voltage of −0.9 V, mobility of 0.884 cm^2^/Vs, on-off ratio of 5.5 × 10^5^, and subthreshold swing of 0.41 V/dec.

## 1. Introduction

Oxide semiconductor, as the active layer for TFTs, has drawn much interest recently owing to their advantageously high carrier mobility, high optical transparency in the visible light region, high thermal/environmental stability, and low process temperature [1,2,3,4,5,6,7]. Among them, ZnO has been regarded as a promising material and is ubiquitous in optoelectronic devices as a result of its low toxicity, abundance on Earth, wide energy bandgap of 3.37 eV, and large exciton-binding energy of 60 meV at room temperature [8,9]. Many research groups have endeavored to develop TFTs with high electrical and optical properties, which can be utilized in organic light emitting diodes (OLEDs) and active matrix liquid crystal displays (AMLCDs). The resistivity of the thin film cannot be too high since it will cause low output current, while it cannot be too low as it will bring about distastefully high leakage current and surface current. Conventional TFT material, such as hydrogenated amorphous silicon (a-Si:H) usually benefit from ambient deposition conditions and ease of fabrication. Nonetheless, such devices exhibit poor mobility [10], which is deemed undesirable for high-speed devices or fast switching circuits.

One way to enhance the mobility is addition of appropriate metal element [11]. Hosono et al. reported that amorphous oxides comprising heavy metal cations with an ns^0^ (*n* ≥ 4) electronic configuration can accomplish elevation of mobility due to high overlapping, large diameter, and high spherical symmetry of ns^0^ orbitals [12,13]. Consequently, indium-doped or tin-doped ZnO materials become attractive and popular. For example, indium zinc oxide (IZO) [14], zinc tin oxide (ZTO) [15], indium zinc tin oxide (IZTO) [16], and indium gallium zinc oxide (IGZO) [17] have been reported in the literature. Compared to the previous studies, obviously there is little research related to incorporation of titanium (Ti). Although IGZO holds the edge and is commonly-seen in TFT fabrication, it is known that scientists have tried to seek alternatives to replace gallium because the weak Ga–O bond may result in instability issues. Like magnesium (Mg), hafnium (Hf), and zirconium (Zr), titanium has the ability to suppress excess carrier generation and make devices more stable under bias and illumination stress [18,19,20]. Furthermore, titanium is non-noxious, and has a lower electronegativity (1.54) as well as a lower standard electrode potential (−1.63 V) compared to those of Zn (1.65 and −0.76 V) [21], which means it is more likely to oxidize than zinc and can be used as a carrier suppressor in ZnO-based TFTs. Accordingly, InTiZnO, as a novel material, is expected to exhibit high optical and electrical performance.

In this paper, we proposed the fabrication of indium titanium zinc oxide (InTiZnO) TFT by utilizing Radio-Frequency (RF) sputtering system. Our goal is to seek whether InTiZnO has possibility of being a promising semiconductor material that can be used for optoelectronic component fabrication. It is known thin films can be grown through spray pyrolysis, chemical vapor deposition, sol–gel, and sputtering methods. Sputtering is preferable because of its large-area deposition, stable growth rate, and good film quality [22]. We focused on the impact of oxygen-related defects on the electrical performance of the device. The compensation level of oxygen-related defects was investigated and discussed.

## 2. Materials and Methods

**Figure 1:**
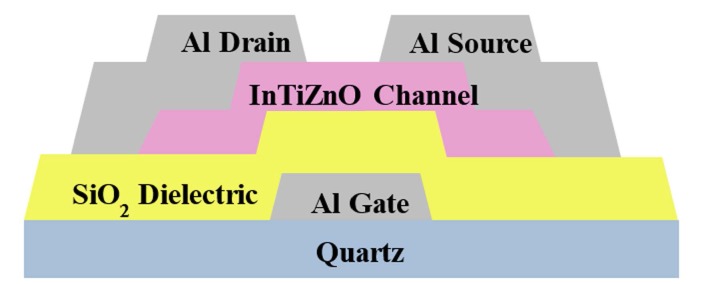
Schematic diagram of the sputtered InTiZnO thin film transistor.

The schematic diagram of the fabricated bottom-gate InTiZnO TFT was shown in Figure 1. First, quartz substrates were chemically cleaned by an ultrasonicator. A 70-nm-thick aluminum was subsequently deposited on the as-cleaned substrate by thermal evaporation with a metal mask. Silica of 200 nm was then grown by plasma-enhanced chemical vapor deposition (PECVD, SAMCO PD-220NA, Kyoto, Japan). The channel layer InTiZnO of 50 nm was sputtered with various oxygen flow ratios. It is noted an InTiZnO target (In:Ti:Zn = 99:1:99 in molar ratio) was utilized, the RF power was 80 W, and the working pressure was 5 mTorr. Sputtering was performed in argon and oxygen ambience at room temperature with manipulated gas flows. Argon flow was from 49 sccm to 45 sccm, while oxygen flow was from 1 sccm to 5 sccm. Next, aluminum of 70 nm was thermally grown to act as source and drain, whose patterns were defined by another metal mask. All processes were done without intentional heating or post-annealing. No etching, lift-off, or other photolithography technique was included. The gate length and gate width of the fabricated TFTs were 100 μm and 1000 μm, respectively. For thin film analysis, we prepared 100-nm-thick InTiZnO on cleaned substrate. Surface morphology of the films was examined via atomic force microscope (AFM, NT-MDT Solver P47-PRO, Moscow, Russia). Transmittance spectrum was recorded via a UV-3101 UV–Vis–NIR spectrophotometer (Shimadzu UV-3101PC, SHIMADZU Corp., Kyoto, Japan). Crystallinity of the films was investigated by X-ray Diffraction (XRD, Rigaku ATX-E, Tokyo, Japan) with a Cu Kα radiation source (λ = 1.54056 Å). X-ray Photoelectron Spectroscopy (XPS, VG ESCALAB220i-XL, Thermo Scientific, Waltham, MA, USA) was applied to analyze chemical state of oxygen. The TFTs were subject to current–voltage (*I*–*V*) characteristics measurement in dark at room temperature by a semiconductor parameter analyzer (Agilent B1500, Palo Alto, CA, USA). 

## 3. Results and Discussion

**Figure 2:**
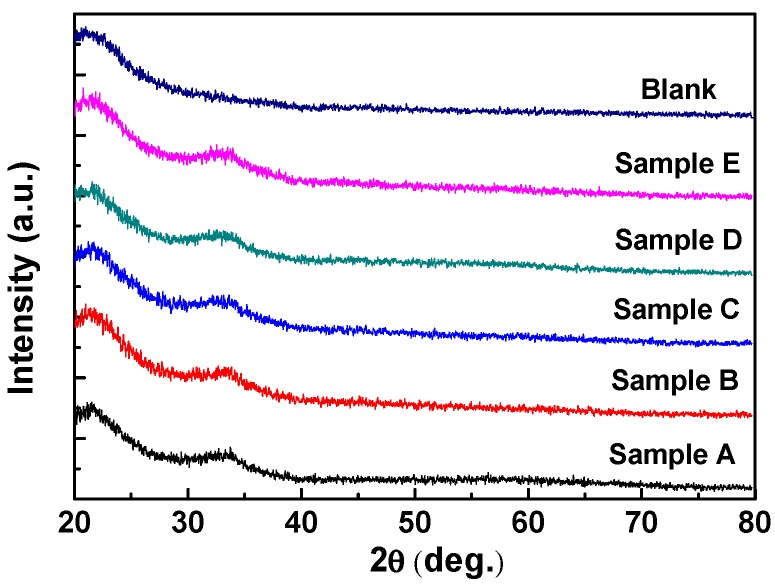
XRD spectrum measured from a blank quartz glass and Samples A, B, C, D, and E.

**Figure 3:**
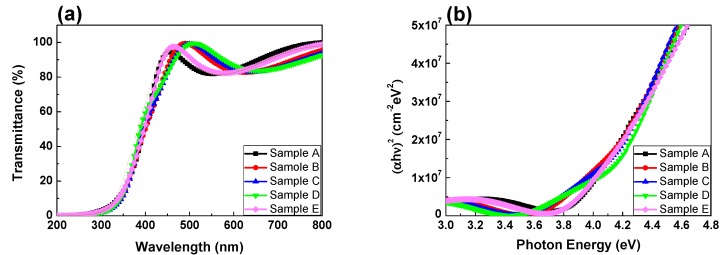
(**a**) Transmittance spectrum measured from Samples A, B, C, D, and E. (**b**) Absorption coefficient versus photon energy for these five samples.

The measured XRD patterns are demonstrated in Figure 2. It is notable that we prepared thin films of 100 nm on quartz substrate for film analyses. Five respective samples were named in light of their oxygen flow ratios during the sputtering process. In other words, Sample A indicated the device with oxygen flow ratio of 2%, Sample B was for 4%, Sample C was for 6%, Sample D was for 8%, and Sample E was for 10%. No evident diffraction peak of crystalline phase was observed from the spectrum, except for the peak at 21.4° to 21.5° attributed to quartz substrate. The result was similar to the report of A. Liu et al. in 2014 [23]. There is a weak peak located at around 32°, which may be attributed to ZnO crystal (JCPDS #890510). However, sputtered quaternary compounds are complex and usually amorphous owing to the nature of deposition method. Consequently, we tend to believe the characterization results revealed that InTiZnO films were amorphous. Figure 3a shows the transmittance of InTiZnO thin films sputtered under various oxygen flow conditions. The variation of oxygen flow ratio had little influence on the absorption edge. It was observed that the average transmittance of each sample was over 80% in the visible region. To go further, the relation between absorption coefficient (α) and the incident photon energy (hν) was plotted, as shown in Figure 3b. It is known the calculation of the corresponding optical bandgap of a certain semiconductor material is described by Tauc’s Law and is given as

$${\left(\alpha h\nu \right)}^{2}\;=\;B\left(h\nu -Eg\right),$$

where B a constant and Eg the bandgap [24]. By estimating the tangent lines from the curves it turned out the calculated optical bandgap of InTiZnO was 3.90–4.06 eV, implicating InTiZnO is a wide-gap material having potential for further photo-sensing applications. It is notable that optical absorption by defects appears at energies lower than the optical gap. Band tailing is speculated to be attributed to the combing effects of impurity disorder and other defects [25,26].

**Figure 4:**
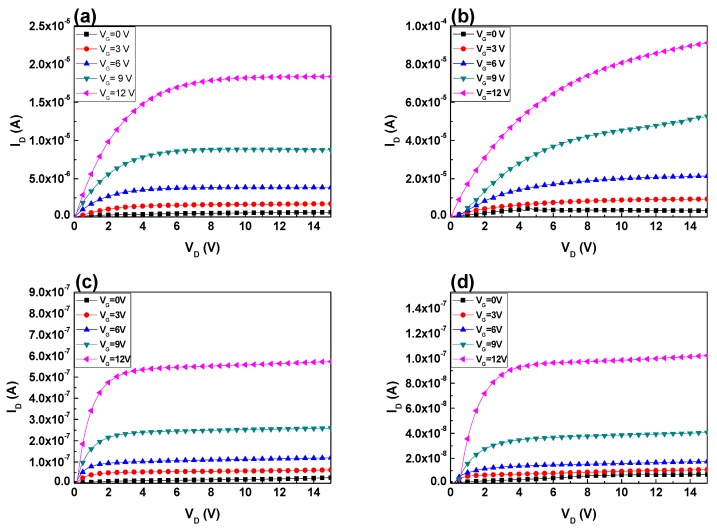

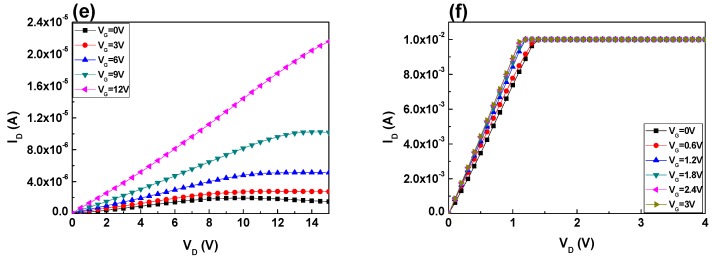
*I*_D_-*V*_D_ of InTiZnO TFTs as a function of oxygen flow ratios: (**a**) Sample A, (**b**) Sample B, (**c**) Sample C, (**d**) Sample D, and (**e**) Sample E, and (**f**) Sample F with 0% oxygen flow ratio.

**Figure 5:**
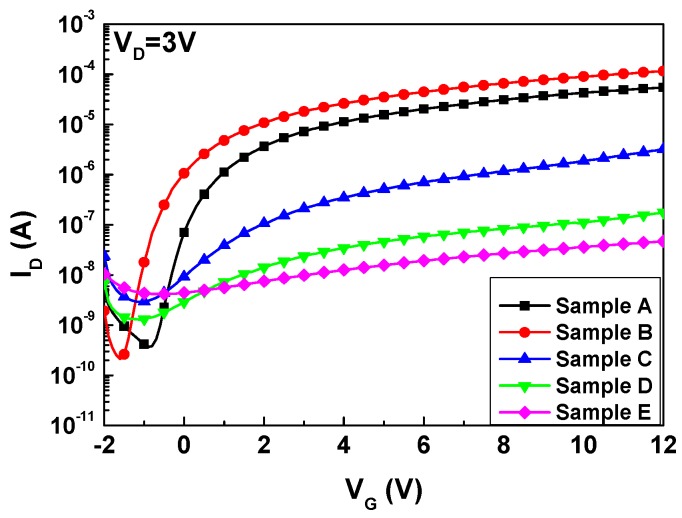
Transfer curve of InTiZnO TFTs with different oxygen flow ratios.

Figure 4a–e shows the output (*I*_D_-*V*_D_) curves of InTiZnO TFTs measured in dark at room temperature. The drain voltage was swept from 0 V to 15 V with a step of 0.1 V, while the gate voltage was from 0 V to 12 V with a step of 3 V. The obvious pinch-off and drain-current saturation suggested the electron transport in the active layers was well controlled by the gate and drain bias. Compared to other samples, Sample E seemed to be a bit worse, which was speculated to stem from excess incorporation of oxygen. It will be discussed later. The transfer curves of InTiZnO TFTs derived in dark at room temperature is shown in Figure 5. The drain voltage was fixed at 3 V while the gate voltage was from −2 V to 12 V with a step of 0.1 V. It can be clearly seen that five samples exhibited typical n-type characteristics. For comparison, the threshold voltage (*V*_T_), mobility (μ_eff_), on-off current ratio, subthreshold swing (*SS*), and total equivalent trap states (*N*_t_) of each sample are listed in Table 1 (Values with error for Samples A, B, and C are shown in Table S1 in Supplementary Materials). By drawing the *I*_D_^1/2^ versus *V*_G_ figure, *V*_T_ could be determined by setting *I*_D_^1/2^ = 0. The device mobility and subthreshold swing are defined as

$$I_{D}=\frac{W}{2L}C_{\mathrm{ox}}\mu_{\mathrm{eff}}{\left(V_{G}-V_{T}\right)}^{2},$$

$$SS=\frac{\partial V_{G}}{\partial \log I_{D}},$$

where $I_{D}$ the drain current, $\frac{W}{L}$ the dimension of the device, *C*_ox_ the gate capacitance per unit area,$\;V_{G}$ is the gate voltage, and *V*_T_ the threshold voltage. It is known that *SS* is related to the interface and bulk trap density of the TFTs. The total equivalent trap states can be acquired by using the following equation [27],

$$N_{t}=\left[\frac{SS\log\left(e\right)}{kT/q}-1\right]\frac{C_{\mathrm{ox}}}{q}\;,$$

where *q* is the charge of the electron, *k* the Boltzmann constant, *T* the temperature, *SS* the subthreshold swing, and *C*_ox_ the gate capacitance per unit area. The dielectric constant of SiO_2_ is 3.9, and the calculated capacitance was about 17 nF/cm^2^_._ It is noteworthy that when sweeping from 12 V to −2 V during *I*_D_-*V*_D_ measurement, another curve is expected to appear, which is related to a reliability issue.

InTiZnO films prepared under various gas mixture revealed a homogeneous surface (images shown in Figure S1 in Supplementary Materials). The grains are shaped as round cones uniformly distributed on the 2 × 2 μm area. From AFM analysis, surface roughness varied as a function of oxygen flow ratio. It was gradually increased from 1.003 nm to 1.354 nm by increasing the oxygen flow. The rougher surface was due to the more drastic ion crashing on the film during sputtering. Hence, higher oxygen flow ratio may cause objectionable surface scattering, leading to mobility reduction. A rougher surface should also be responsible for the variation of *N*_t_.

On the other hand, it is known that oxygen vacancy acts as a carrier provider in ZnO-based materials. When oxygen flow ratio increased, the amount of oxygen vacancies in the thin film reduced. It can be seen that free carriers became less from the *I*–*V* measurement. In *I*_D_-*V*_D_ measurement, samples with a higher oxygen flow ratio would reach a lower saturation current. The saturation current: Sample D < Sample C < Sample B. In the *I*_D_-*V*_G_ transfer curve, the on current at 12 V: Sample E < Sample D < Sample C < Sample B. Accordingly, it is confirmed that concentration of carriers was suppressed. However, participation of excess oxygen during film growth could compromise the performance of devices. Namely, it would bring about undesirable oxygen-related deficiencies and make TFTs difficult to operate desirably. Augmentation in trap states due to increasing oxygen flow ratio could hinder the carriers in the channel layer from smooth transport. Therefore, the mobility decreased from 1.625 cm^2^/Vs to 0.004 cm^2^/Vs and subthreshold swing increased from 0.32 V/dec to 8.57 V/dec. It is notable that our devices with a negative threshold voltage were in depletion mode (D-mode). For a depletion-mode transistor, the device is normally-on at zero gate–source voltage. It indicates that there may be a power consumption issue, which could be solved by increasing the thickness of dielectric layer or replacing high-k material with the original gate oxide.

**Figure 6:**
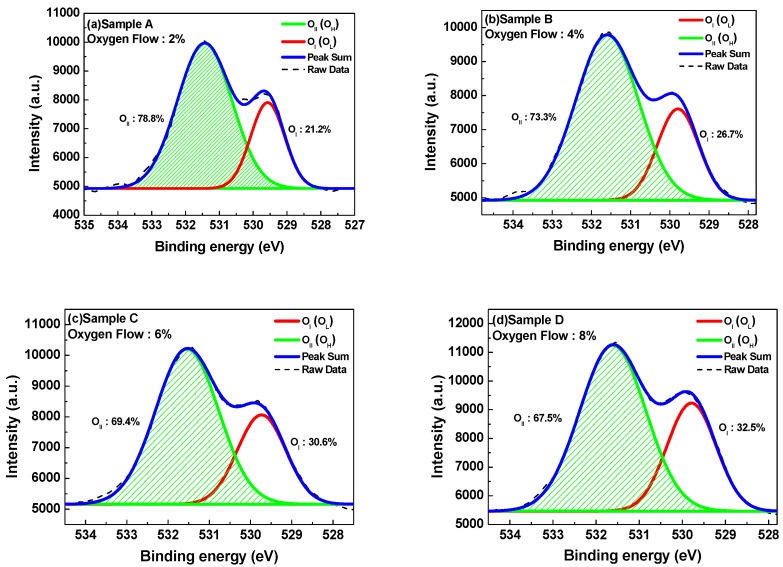

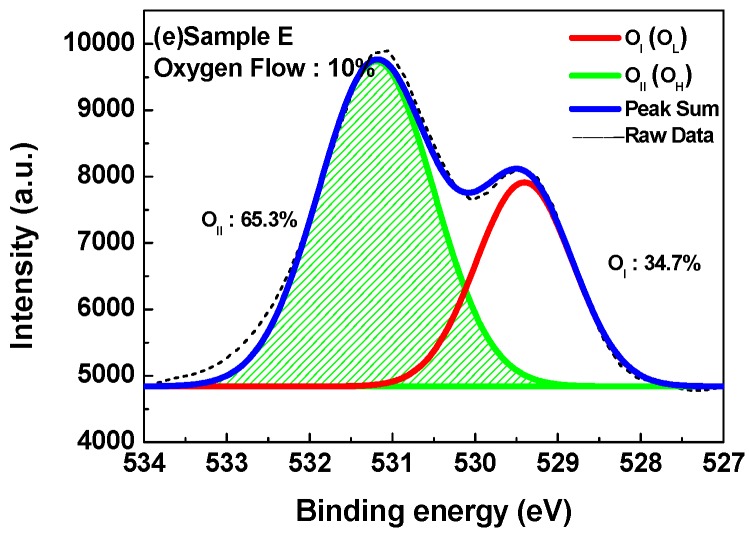
XPS O 1s spectra of InTiZnO thin films with various oxygen flow ratios: (**a**) Sample A, (**b**) Sample B, (**c**) Sample C, (**d**) Sample D, and (**e**) Sample E.

Figure 6a–e is XPS spectra of O 1s of InTiZnO films grown with different oxygen flow ratios. The spectra were deconvoluted into two peaks by Gaussian fitting: O_I_ occurred at 529.6 eV was assigned to O^2−^ species in the lattice, and O_II_ located at 531.3 eV was associated with oxygen vacancies or defects, or O^2−^ ion in oxygen-deficient region [8,28]. By evaluating peak area O_II_ over (O_I_ + O_II_) it can be seen the proportion decreased from 78.8% to 65.3% as the oxygen flow ratio increased from 2% to 10%. Assuming small contributions of the film surfaces, the result indicated that concentration of oxygen vacancy in the films was reduced in terms of proportion with the increasing oxygen flow ratio, and it was consistent with the device performance. That is, manipulation of oxygen flow and argon flow is a performance trade-off issue. On the basis of the electric properties, we deduced that Sample A and Sample B were superior to other samples, whereas on-off current ratio of Sample B was higher than that of Sample A. It implied the gate control ability of Sample B was better, as a result of well-manipulated oxygen flow ratio of 4%. Sample B was considered to be the optimal device in this work, exhibiting a mobility of 0.884 cm^2^/Vs, on-off ratio of 5.5 × 10^5^, and subthreshold swing of 0.41. Table 2 lists previous studies similar to InTiZnO. It can be seen that our devices are competent.

On the other hand, sample without oxygen (oxygen flow ratio = 0%, only pure argon) was also prepared. During *I*–*V* measurement, the applied drain voltage swept from 0 V to 4 V with an increment of 0.1 V, while the gate voltage was from 0 V to 3 V with an increment of 0.6 V. In Figure 4f, it was observed the output current reached the compliance current (10 mA) of the semiconductor parameter analyzer. The result indicated that the InTiZnO 0% TFT was over-conductive. The innate defects from ZnO-based material were not suitably compensated, lacking feasibility of device operation.

## 4. Conclusions

In summary, we reported the fabrication of InTiZnO TFTs. The transmittance of InTiZnO was more than 80% in the visible region. The energy bandgap of InTiZnO was derived to be 3.90–4.06 eV. The results showed InTiZnO had great potential for UV sensors. By manipulating the oxygen flow ratio during sputtering, oxygen vacancies could be filled as confirmed by XPS measurement, making it possible to prepare the optimal InTiZnO TFTs that exhibited high electrical performance. If only argon took part in growth of active layers, excess carriers would exist, resulting in high leakage currents. We found that 4% oxygen flow ratio was preferable. Under such conditions, Sample B showed a threshold voltage of −0.9 V, mobility of 0.884 cm^2^/Vs, on-off ratio of 5.5 × 10^5^, and subthreshold swing of 0.41 V/dec. We believe the findings reveal a great step toward knowledge of the quaternary semiconductor material InTiZnO. The combination of transparency, ease of fabrication under room temperature, and high on-off current ratio makes InTiZnO TFT very promising for the next-generation optoelectronic device.

## Figures and Tables

**Table 1:** Electronic parameters of each unannealed InTiZnO TFT measured in dark at room temperature.

Samples	Oxygen Flow Ratio	*V*_T_ (V)	μ_eff_ (cm^2^/Vs)	On-Off Current Ratio	*SS* (V/dec)	*N*_t_ (cm^−2^)
Sample A	2%	−0.35	1.625	1.5 × 10^5^	0.32	4.6 × 10^11^
Sample B	4%	−0.9	0.884	5.5 × 10^5^	0.41	6.2 × 10^11^
Sample C	6%	−0.5	0.235	1.1 × 10^3^	1.62	2.8 × 10^12^
Sample D	8%	−1.4	0.006	1.4 × 10^2^	2.46	4.3 × 10^12^
Sample E	10%	−4	0.004	1.1 × 10^1^	8.57	1.5 × 10^13^

**Table 2:** Electrical performance of ZnO-based TFTs reported in the literature

Materials	Deposition Method	*V*_T_ (V)	μ_eff_ (cm^2^/Vs)	On-Off Current Ratio	*SS* (V/dec)	*N*_t_ (cm^−2^)
InZnO [29]	Sol–gel	0.18	0.15	10^5^	0.86	N.A.
InMgZnO [30]	Sol–gel	N.A.	0.56	<10^5^	2.2	N.A.
InTiZnO [31]	Sol–gel	8.49	0.04	10^4^	1.06	N.A
InTiZnO [24]	PLD	7.89	2.58	10^8^	0.76	1.5 × 10^12^
InTiZnO, Sample A (this work)	sputter	−0.35	1.625	1.5 × 10^5^	0.32	5.7 × 10^11^
InTiZnO, Sample B (this work)	sputter	−0.9	0.884	5.5 × 10^5^	0.41	7.3 × 10^11^

## Supplementary Materials

The following are available online at www.mdpi.com/2079-4991/7/7/156/s1, Figure S1: AFM images of each InTiZnO samples, Table S1: Electronic parameters for five Samples, with errors for Samples A, B, and C.
