# Supplementary material for: Oxygen Partial Pressure Impact on Characteristics of Indium Titanium Zinc Oxide Thin Film Transistor Fabricated via RF Sputtering
Source: Nanomaterials (Basel). 2017 Jun 26;7(7):156. doi: 10.3390/nano7070156 (PMC5535222; doi:10.3390/nano7070156)
Supplement: Supplementary file 1 [file nanomaterials-07-00156-s001.pdf]

Supplementary Materials: Oxygen Partial Pressure Impact on Characteristics of Indium Titanium Zinc Oxide Thin Film Transistor Fabricated via RF Sputtering

Ming-Hung Hsu, Sheng-Po Chang *, Shoou-Jinn Chang, Wei-Ting Wu, and Jyun-Yi Li

Institute of Microelectronics & Department of Electrical Engineering Center for Micro/Nano Science and Technology Advanced Optoelectronic Technology Center, National Cheng Kung University, Tainan 701, Taiwan; hsuminghung0121@gmail.com (M. H. Hsu); changsj@mail.ncku.edu.tw (S. J. Chang); waiting31317@gmail.com (W. T. Wu); z823040@gmail.com (J. Y. Li)

* Correspondence: changsp@mail.ncku.edu.tw; Tel.: +886-6-275-7575 (ext. 62400-1208)

This section includes: Figure S1, Table S1.

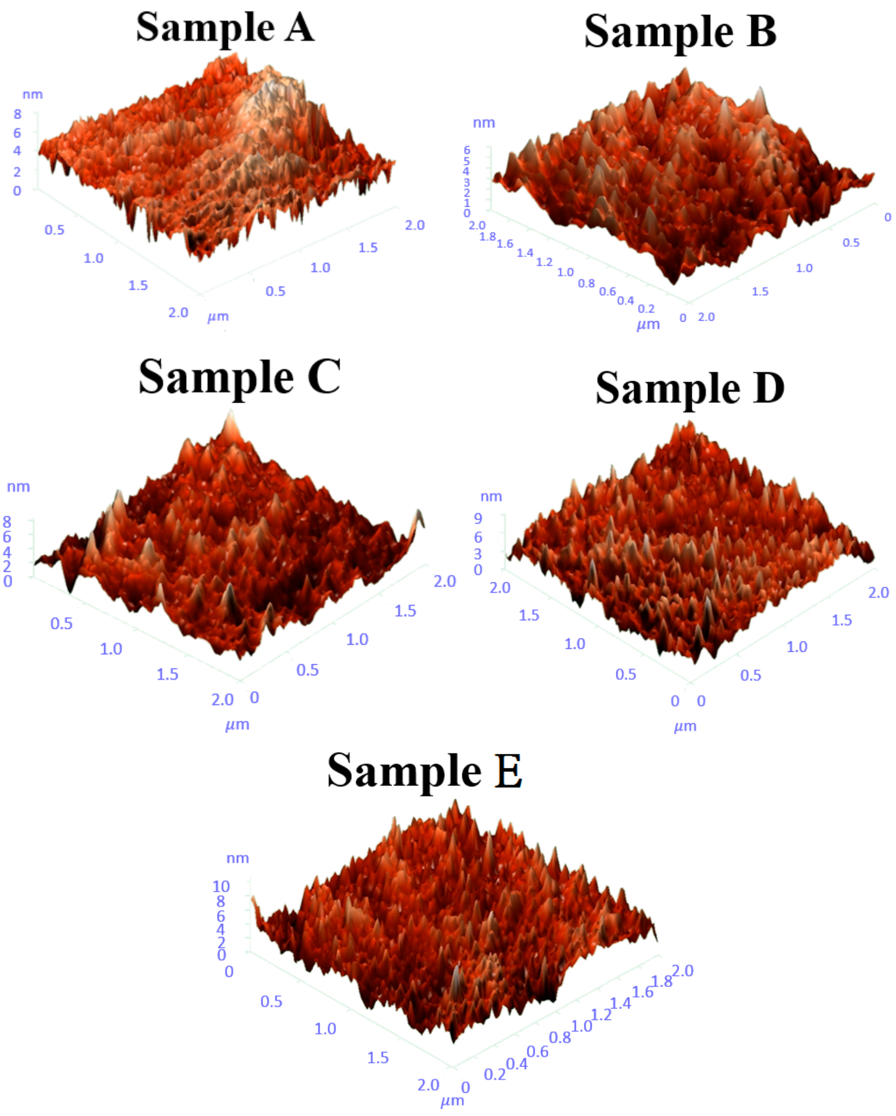

Figure S1. AFM images of each InTiZnO samples.

Table S1. Electronic parameters for five Samples, with errors for Sample A, B, and C.

**Samples**	V_T **(V)**	μ_{eff} (cm^2/Vs)	**On-off current ratio**	**SS (V/dec)**	N_t **(10^{11} cm^{-2})**
Sample A (2%)	-0.35±0.1	1.625±0.001	1.5×10^5	0.320±0.003	4.6±0.1
Sample B (4%)	-0.9±0.01	0.884±0.003	5.5×10^5	0.410±0.002	6.2±0.1
Sample C (6%)	-0.5±0.03	0.235±0.001	1.1×10^3	1.62±0.04	28±1
Sample D (8%)	-1.4	0.006	1.4×10^2	2.46	43
Sample E (10%)	-4	0.004	1.1×10^1	8.57	150
